# Culture of Methanogenic Archaea from Human Colostrum and Milk

**DOI:** 10.1038/s41598-019-54759-x

**Published:** 2019-12-09

**Authors:** Amadou Hamidou Togo, Ghiles Grine, Saber Khelaifia, Clotilde des Robert, Véronique Brevaut, Aurelia Caputo, Emeline Baptiste, Marion Bonnet, Anthony Levasseur, Michel Drancourt, Matthieu Million, Didier Raoult

**Affiliations:** 1 IHU-Méditerranée Infection, Marseille, France; 2 Aix Marseille Univ, IRD, APHM, MEPHI, Marseille, France; 3 APHM, CHU Hôpital de la Conception, Service de médecine néonatale, F-13385 Marseille, France; 4 APHM, CHU Hôpital Nord, Service de médecine néonatale, Marseille, France

**Keywords:** Archaeal genomics, Archaeal physiology

## Abstract

Archaeal sequences have been detected in human colostrum and milk, but no studies have determined whether living archaea are present in either of these fluids. Methanogenic archaea are neglected since they are not detected by usual molecular and culture methods. By using improved DNA detection protocols and microbial culture techniques associated with antioxidants previously developed in our center, we investigated the presence of methanogenic archaea using culture and specific *Methanobrevibacter smithii* and *Methanobrevibacter oralis* real-time PCR in human colostrum and milk. *M*. *smithii* was isolated from 3 colostrum and 5 milk (day 10) samples. *M*. *oralis* was isolated from 1 milk sample. For 2 strains, the genome was sequenced, and the rhizome was similar to that of strains previously isolated from the human mouth and gut. *M*. *smithii* was detected in the colostrum or milk of 5/13 (38%) and 37/127 (29%) mothers by culture and qPCR, respectively. The different distribution of maternal body mass index according to the detection of *M*. *smithii* suggested an association with maternal metabolic phenotype. *M*. *oralis* was not detected by molecular methods. Our results suggest that breastfeeding may contribute to the vertical transmission of these microorganisms and may be essential to seed the infant’s microbiota with these neglected critical commensals from the first hour of life.

## Introduction

**Figure 1:**
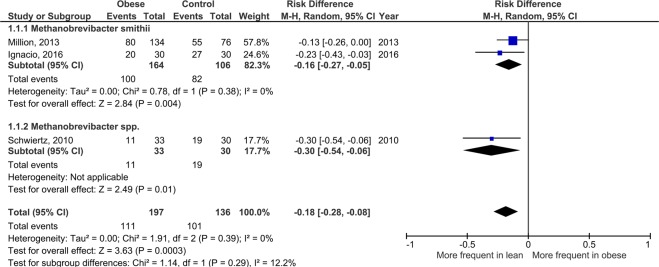
Meta-analysis comparing the frequency of *Methanobrevibacter* species in human feces from obese and lean individuals. The study of Million *et al*.^52^ included individuals from previous studies from the same center^51,68^. The study of Ignacio *et al*. was performed in Brazil and confirmed the decreased frequency of *M*. *smithii* in obesity^61^. No substantial heterogeneity was observed between the 2 studies (I^2^ = 0%), with a highly significant association (*p* = 0.004). These results were consistent with those reported by Scwhiertz *et al*. focusing on *Methanobrevibacter* species^53^ with very low heterogeneity (I^2^ = 12%) suggesting a consistent and significant (*p* = 0.0003) effect at the genus level. Studies with a lower taxonomical resolution did not show any consistent result. For instance, studies at the *Methanobacteriales* order level included *M*. *stadtmanae*^15^ associated with proinflammatory properties *in vitro*^46^ and in clinical studies^64^. These results suggest a genus-specific effect of human methanogenic Archaea on weight regulation and obesity and support that *M*. *smithii* is a neglected critical commensal for human health.

Breastfeeding is a major determinant of human health^1^. Breast colostrum and milk contain a very diverse bacterial microbiota that plays a key role in human health^2^. In this context, while archaeal sequences have been detected^3,4^, no studies have determined whether living archaea are present in human milk^2^. The human archaeome is increasingly recognized owing to dedicated molecular methods^5^. The genomic analysis of *Methanobrevibacter smithii*^6^, the main human methanogenic archaea, showed an evolutive adaptation to the human gut^7^. The emerging role of this species is notable for methanogenic archaea^8^, which are particularly adapted to the gut and key components of human-archaeal-bacterial mutualism through methanogenesis^9^. Methanogenesis improves energy harvest by consuming end products of microbial fermentation^7,9^. The role for human health and metabolism is supported by the fact that *M*. *smithii* was found in virtually all healthy lean human adults but was depleted in obese individuals (Fig. 1)^10,11^ and in those with severe acute malnutrition^12^.

Methanogenic archaea form a co-occurrence network with other heritable Bacteria such as the family *Christensenellaceae*^10,13^, and the abundances of both are enriched in lean healthy individuals^10^. However, the way(s) of vertical transmission of methanogenic Archaea remain a missing piece in the puzzle of metabolic phenotype inheritance^2^. Indeed, while both the human genome and microbiome are inherited^10^, the microbiome has been found to be more informative than the human genome for predicting obesity^10^. In this context, we previously showed that *M*. *smithii* consistently colonizes the newborn stomach, suggesting an early gut colonization^14^. Accordingly, the culture of methanogenic archaea from human colostrum and milk represents an exciting challenge.

Human methanogenic archaea remain largely neglected, and their role in human health is underestimated for several reasons^15^. First, the culture of methanogenic archaea was, until recently, very tedious, expensive, and time-consuming. Second, metagenomics and 16S amplicon sequencing studies neglected human methanogenic Archaea due to DNA extraction bias, depth bias (majority species are more likely to be detected) and primer bias (specifically for 16S amplicon sequencing studies)^5^. This is supported by the fact that *M*. *smithii* is detected in 64% of human adults by 16S amplicon sequencing^10^ but in 96% by specific PCR^6^. Third, a large part of the current knowledge on the human microbiome relies on transplantation studies in germ-free mice^10^. Strikingly, *M*. *smithii* does not persist in mice^10^.

As a result, only a few studies have reported the culture of methanogenic archaea from humans (see Supplementary Tables S1 and S2)^13,14,16–34^. The main limitation of culture is that most human gut microbes including methanogenic archaea are extremely sensitive to oxygen and its reactive derivatives. In this context, we recently established a microbial culture technique using a patented antioxidant mixture containing high doses of ascorbic acid, glutathione^35^ and uric acid^36–38^ (see conflict of interest section). We then aerobically cultured several methanogenic archaeal strains from culture collections using a dual chamber system. All methanogenic archaea tested could be cultured^29^. This new antioxidant-based culture system allowed us to investigate the presence of methanogenic archaea in human colostrum and milk by culture and genome sequencing. We also used specific PCR (qPCR) as a confirmatory method and compared the characteristics of the mother and child according to the detection of *M*. *smithii* in colostrum and/or milk, identified in this study as the most prevalent methanogenic archaea in human colostrum and milk.

## Results

### Included mothers

A total of 128 mothers who had provided at least 1 sample (colostrum and/or milk (day 10)) were included; 138 samples including 118 colostrum and 20 milk samples were obtained and analyzed by at least one technique (culture and qPCR). The full clinical details and results are provided in Dataset 1. Among the 128 included mothers, 14 (11%) were obese before pregnancy, 33 (26%) were overweight, 74 (58%) were lean and 7 (5%) were underweight.

### Culture and isolation

For the culture of methanogenic archaea, 20 samples from 13 mothers were analyzed (Table 1 and Dataset 1). The culture was positive in 9 out of 20 samples, including 3 colostrum and 6 milk samples. *M*. *smithii* was isolated from 8 samples corresponding to 5 mothers (with both colostrum and milk isolation in 3 mothers). Focusing on the 6 mothers for whom both colostrum and milk (day 10) were available, the excretion of *M*. *smithii* in colostrum was associated in all cases with excretion in milk on day 10 (3/3). In contrast, the three mothers who produced culture-negative colostrum also produced culture-negative milk. For all culture-positive samples for methanogens, colonies appeared after 9 days of incubation (Supplementary Fig. 1). *M*. *oralis* was isolated from 1 sample (milk, no colostrum available from this mother).

**Table 1:** Summary of the results of the molecular and culture analyses.

	Colostrum		Milk (around day 10)	
	Methane production	Strain identification	Methane production	Strain identification
Mother_018	NA	NA	−	ND
Mother_076	−	ND	+	***M***. ***oralis*** **strain M2 CSUR P5920**^**a**^
Mother_095	+	***M***. ***smithii*** **strain C1 CSUR P5920**	+	***M***. ***smithii*** **strain M5 CSUR P5919**
Mother_096	+	***M***. ***smithii*** **strain C2 CSUR P5816**^**a**^	+	***M***. ***smithii*** **strain M6 CSUR P5818**
Mother_097	+	***M***. ***smithii*** **strain C3 CSUR P5922**	+	***M***. ***smithii*** **strain M7 CSUR P5820**
Mother_098	−	ND	−	NA
Mother_099	−	ND	ND	ND
Mother_100	−	ND	ND	ND
Mother_101	−	ND	ND	ND
Mother_102	−	ND	−	NA
Mother_103	−	ND	−	NA
Mother_104	NA	ND	+	***M***. ***smithii*** **strain M1 CSUR P5819**
Mother_105	NA	ND	+	***M***. ***smithii*** **strain M3 CSUR P5921**

Methane production detected by gas chromatography from a Hungate tube with SAB medium and antioxidants inoculated with maternal colostrum or milk after transport in Ae-Ana medium supplemented with antioxidants. PCR: Polymerase chain reaction detecting all archaea performed on colonies identified on Petri dishes from methane-positive samples. Strain identification was performed by 16S rRNA gene sequencing., +=positive, −=negative, ND: not done, NA: sample not available. ^a^Strains sequenced for genome analysis.

For all 9 strains (8 *M*. *smithii* and 1 *M*. *oralis*), we determined the 16S rRNA gene sequence. For 2 strains, namely, *M*. *smithii* strain C2 CSUR P5816 (isolated from the colostrum of Mother_096) and *M*. *oralis* strain M2 CSUR P5920 (isolated from the milk of Mother_076 on day 10), the 16S rRNA gene sequence was extracted from the genome sequence (16S and genome sequences deposited in a public repository, see Data availability). For the 7 other *M*. *smithii* strains, a partial 16S rRNA gene sequence was obtained (see methods) and deposited in a public repository (see Data availability). All 9 sequences were aligned and compared with the reference sequence of the type strain for each species.

According to the list of prokaryotic names with standing in nomenclature (www.bacterio.net), the reference sequence U55233 of the type strain of *M*. *smithii* (PS = ATCC 35061 = DSM 861 = OCM 144) was used to align and compare the sequences of our 8 *M*. *smithii* strains (Table 2). The sequences of 2 strains (M2 and M6) were identical (100% identity), 3 strains (C1, C3, M3) showed 1 mismatch (99.83%) and 3 strains (M1, M5, M7) showed 2 mismatches (99.61 to 99.66%). The sequence HE654003.1 of the *M*. *oralis* type strain (DSM 7256) was used to align and compare the sequence of our *M*. *oralis* strain. A 99.39% similarity was found with 9 mismatches (1462/1471) and 3 gaps (3/1471 (0.20%)).

**Table 2:** Alignment of the 16S rRNA gene sequences of 8 *M*. *smithii* strains isolated from human colostrum and milk with the reference sequence of the type strain *M*. *smithii* PS (=ATCC 35061 = DSM 861 = OCM 144) - Sequence Accession No. U55233.

Strain	Origin	Sequence number	Length	Identities	Gaps
C2 CSUR P5816	Colostrum	LR590664	1472 bp	1343/1343 (100%)	0/1343 (0%)
M6 CSUR P5818	Milk (day 10)	LR584037	592 bp	592/592 (100%)	0/592 (0%)
M3 CSUR P5921	Milk (day 10)	LR584041	608 bp	604/605 (99.83%)	1/605 (0.16%)
C1 CSUR P5920	Colostrum	LR584035	604 bp	603/604 (99.83%)	0/604 (0%)
C3 CSUR P5922	Colostrum	LR584038	594 bp	593/594 (99.83%)	1/594 (0.17%)
M5 CSUR P5919	Milk (day 10)	LR584040	607 bp	603/605 (99.66%)	2/605 (0.33%)
M7 CSUR P5820	Milk (day 10)	LR584039	597 bp	595/597 (99.66%)	2/597 (0.33%)
M1 CSUR P5819	Milk (day 10)	LR584036	517 bp	516/518 (99.61%)	2/518 (0.38%)

Bp: base pairs.

**Figure 2:**
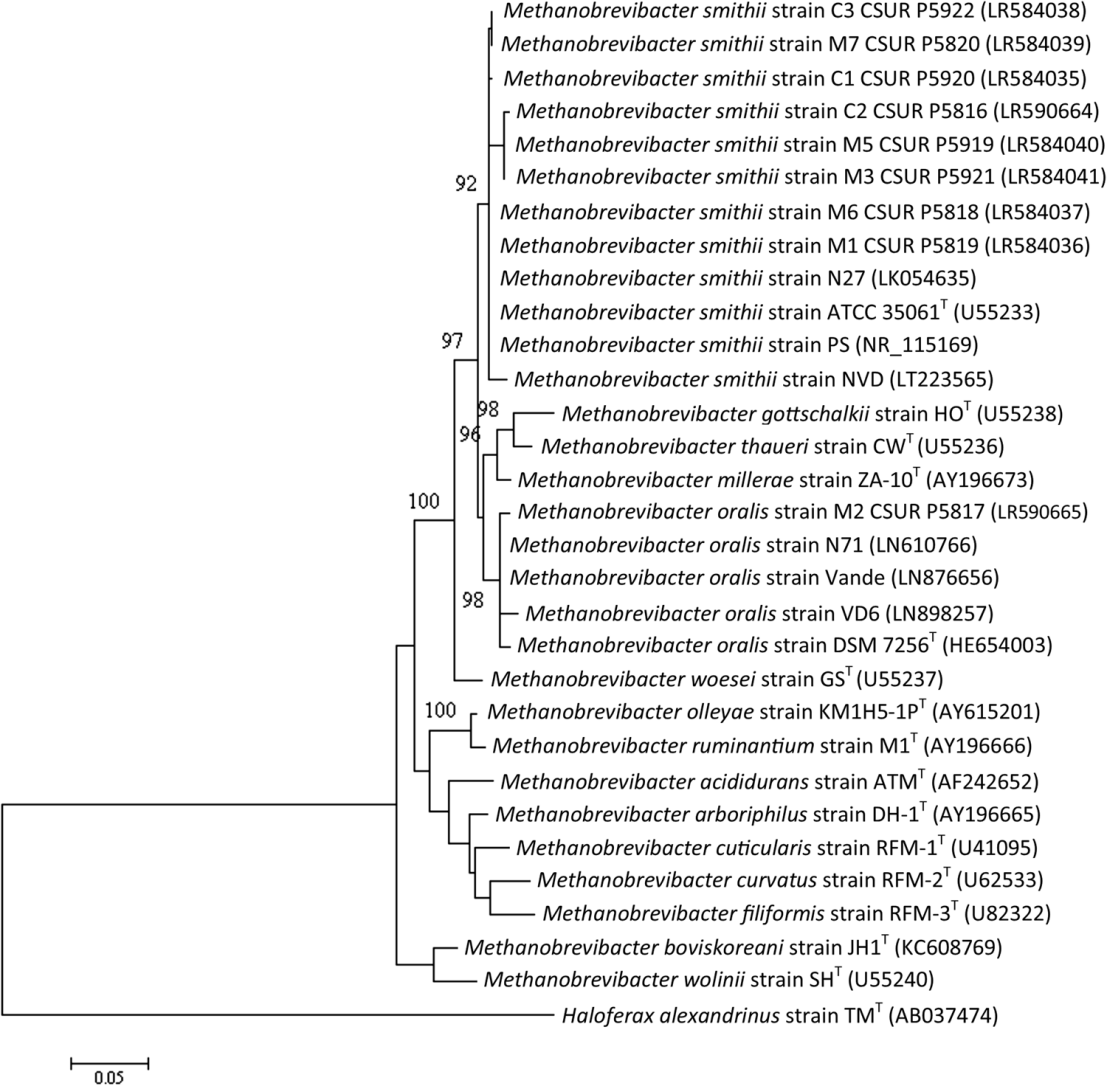
Molecular phylogenetic analysis by maximum likelihood method of the new isolates and their closest species. Bootstrap values ≥90% indicated at nodes. The evolutionary history was inferred by using the maximum likelihood method based on the Kimura 2-parameter model^69^. The tree with the highest log likelihood (−3941.91) is shown. The percentage of trees in which the associated taxa clustered together is shown next to the branches. The initial tree for the heuristic search was obtained automatically by applying the maximum parsimony method. A discrete gamma distribution was used to model the evolutionary rate differences among sites (5 categories (+G, parameter = 0.3801)). The tree is drawn to scale, with branch lengths measured according to the number of substitutions per site. The analysis involved 18 nucleotide sequences. There were a total of 1490 positions in the final dataset. Evolutionary analyses were conducted in MEGA7^70^.

Phylogenetic analysis of the 16S rRNA gene sequence confirmed that all isolated *M*. *smithii* strains grouped together with the type strain and strains previously described and did not form a distinct cluster (Fig. 2). This result suggests that the methanogenic archaeal strains of human colostrum and milk are not different from strains previously isolated, notably from the human gut.

### Genome sequencing

**Figure 3:**
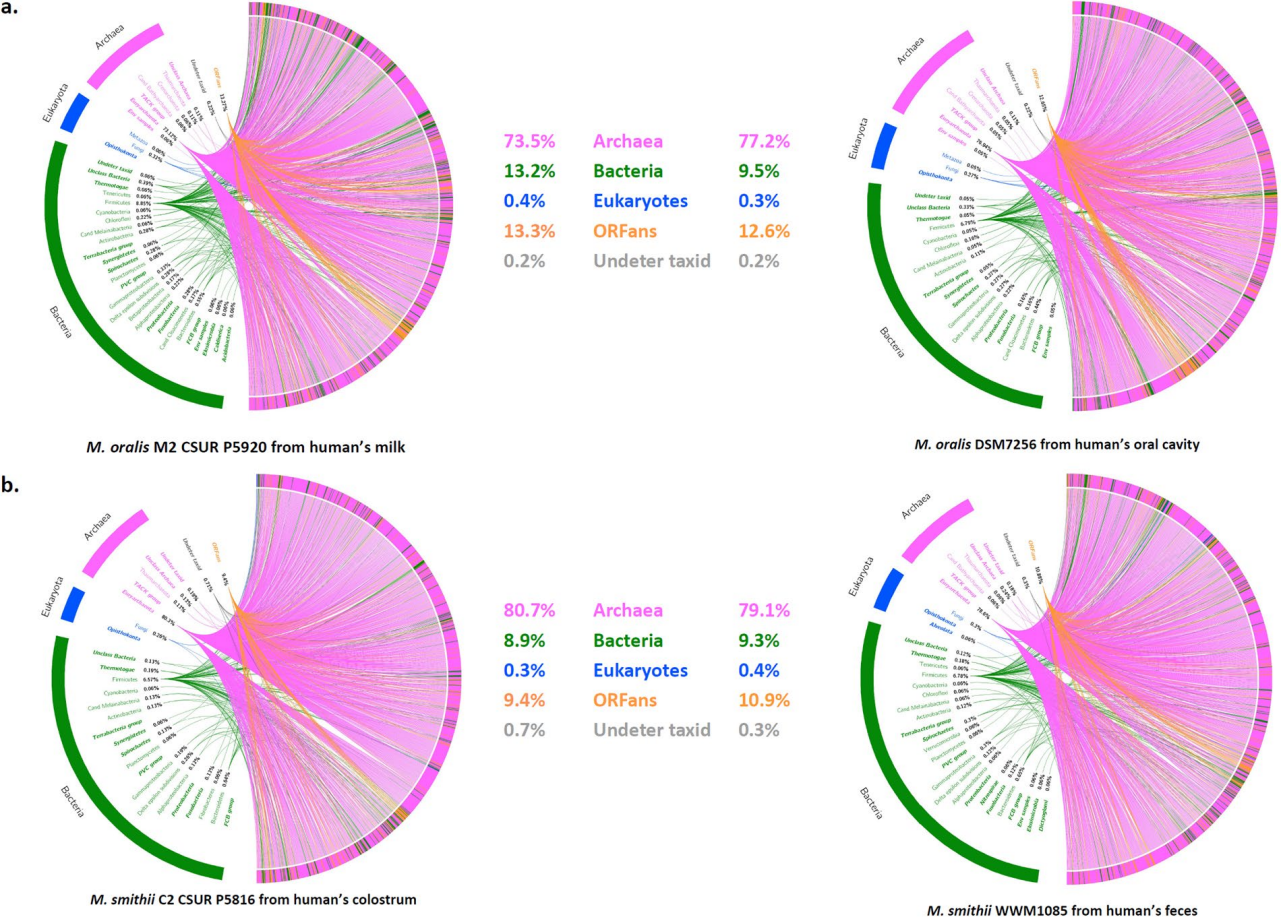
Comparison of rhizomes of *Methanobrevibacter smithii* and *Methanobrevibacter oralis* isolated from human milk with archaea previously described in the digestive tract (*M*. *smithii*) and mouth (*M*. *oralis*). *M*. *smithii* strain C2 CSUR P5816 was isolated from the colostrum of mother_2 (Table 1). Its genome (GenBank number: SAMEA104570327) was compared to the genome of a strain isolated from human feces (=WWM1085, GenBank Number: NQLD000000000000). *M*. *oralis* strain M2 CSUR was isolated from the milk (day 10 after delivery) of mother_11. Its genome P5920 (GenBank Number: SAMEA10457076) was compared to the genome of the type strain of *M*. *oralis* strain ZR (Genome Number: NZ_LWMU00000000.1) isolated from the human oral cavity (=DSM7256, =JCM 30027).

For two strains, *M*. *smithii* strain C2 (colostrum) and *M*. *oralis* strain M2 (milk, Table 1), the genome was sequenced and analyzed. To clarify whether these strains were similar to the human digestive (*M*. *smithii*) or oral (*M*. *oralis*) strains, we analyzed the rhizome as previously described^39^. Rhizome analysis evaluates possible sequence exchanges and their phylogenetic origin. The hypothesis is that strains living in the same microbial environment (human microbiota) share the same lateral sequence exchange profile as phylogenetic groups present in the same ecological niche. Rhizome analysis by visual examination of the global pattern of sequences shared with other prokaryotic species showed that these strains’ profiles were similar to those of *M*. *oralis* and *M*. *smithii* strains previously isolated from the human mouth and intestine (Fig. 3).

### Detection of *M*. *smithii* and *M*. *oralis* using real-time PCR (rt-PCR)

We performed specific *M*. *smithii* and *M*. *oralis* real-time PCR for 127 of the 128 healthy mothers included in the study. In total, 136 samples collected from these 127 mothers were analyzed by real-time PCR and included 117 colostrum and 19 milk samples. Thirty-two of 117 (27.3%) colostrum and 5/19 (26.3%) milk samples were positive for *M*. *smithii*, totaling 37 positive samples from 136 total samples (27.2%). Among the 37 positive samples, the cycle threshold was relatively high (median 38.40, interquartile range [36.75–40.00], range 31.50–40.80). Using calibration curves, we estimated the concentration and found that the abundance was very low (median, 463 copies DNA/mL for colostrum, 339 copies DNA/mL for milk at day 10, Supplementary Fig. 2). Among the 127 mothers included in this analysis, 37 were positive for *M*. *smithii* in colostrum and/or milk (29.1%). *M*. *oralis*-specific real-time PCR was negative on all 136 tested samples (127 mothers).

### Comparison of clinical characteristics of mothers and newborns according to the detection of *M*. *smithii* in colostrum and/or milk

Because only two species were found by culture and *M*. *oralis* was detected solely by culture in only one milk sample, we focused on *M*. *smithii* to uncover associations between the detection of this species and clinical variables. Among the 128 mothers included in the study, 40 (31%) were positive for *M*. *smithii* by culture and/or qPCR. This result suggests that, as previously reported in the gut^6,10^, *M*. *smithii* is the most prevalent methanogenic archaea in human colostrum and milk.

The frequency of the detection of *M*. *smithii* by culture and/or PCR was lower in obese (14%) mothers than in overweight (45%), lean (28%) or underweight (28%) mothers, suggesting an association with maternal metabolic phenotype. The difference between obese (2/14 (14%)) and nonobese mothers (38/114 (33%)) was substantial but not significant (Odds ratio 0.33, 95% confidence interval 0.049–1.41, p = 0.08). Conversely, *M*. *smithii* was more frequent in overweight (45%) than in lean (28%) mothers (Odds ratio 2.09, 95% confidence interval 0.88–4.95, p = 0.047). No other clinical variables appeared to be associated with the detection of *M*. *smithii* in the colostrum or milk (Supplementary Table 3).

**Figure 4:**
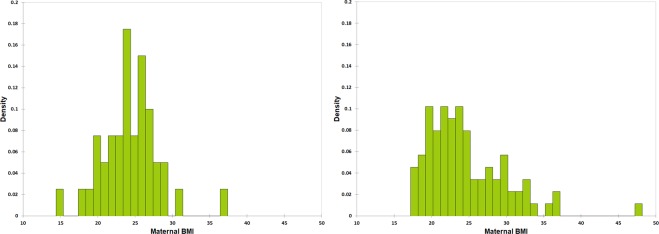
Distribution of maternal BMI values according to the detection of *M*. *smithii* in colostrum and/or milk. Density histograms of pre-pregnancy maternal BMI values for mothers with (left) or without (right) detection of *M*. *smithii* in colostrum and/or milk.

Focusing on the distribution of pre-pregnancy maternal BMI according to the detection of *M*. *smithii* in colostrum and milk (Fig. 4), we observed that the median of the two groups were similar (24.0 and 23.3 for mother with or without detection of *M*. *smithii*). However, observation of the plots and Kolmogorov-Smirnov test (KS test) suggested that the distribution of BMI values was normal in mothers with detection of *M*. *smithii* (KS test p-value > 0.10) but not in mothers without detection of *M*. *smithii* (p < 0.0001). The variances were significantly different (Levene’s test, p = 0.049). Skewness was higher in mothers without detection of *M*. *smithii* (1.38 versus 0.50 in mothers with detection of *M*. *smithii*).

## Discussion

Here, we have shown that methanogenic archaea are alive in colostrum and human milk. Two species have been identified, namely, *M*. *smithii* and *M*. *oralis*, and their presence was demonstrated by culture and confirmed by genome sequencing. Approximately one-third of the mothers harbored *M*. *smithii* in their colostrum and/or milk. Our results are consistent with early neonatal digestive colonization^6,14,40–42^ and the association between gut colonization and organic dairy consumption in children^43^.

The very low median abundance of *M*. *smithii* observed in this study (2 log 10 copies DNA/mL) suggests that minority microbes are important in the human microbiome, particularly in human colostrum and milk. We previously described this as the “minority paradigm^44^”. Moreover, primer bias is another critical limitation of the 16S amplicon sequencing in deciphering the human archaeome^5,45^.

Human methanogenic archaea are critical for human health. The 3 main steps of human digestion are hydrolysis, fermentation and methanogenesis. Fermentation by gut anaerobic bacteria yields short-chain fatty acids (mainly acetate, propionate and butyrate), formate, ethanol, and lactic acid but particularly dihydrogen (H_2_) and carbon dioxide (CO_2_). However, according to the Le Chatelier principle, formate and hydrogen consumption by methanogenesis is critical to accelerate the production of ATP and short-chain fatty acids, thus increasing the efficiency of the energy harvest for the host^9,13^. According to current scientific knowledge, no other microbes or human cells in the human gut are able to replace archaea for methanogenesis, so these symbionts can be considered critical for human health^15^. The very low H_2_-utilization threshold of *M*. *smithii* compared to that of acetogens makes it more efficient at depleting H_2_ from the gut environment^13^.

*M*. *smithii* is the dominant human gut-associated archaeon^13^, is almost ubiquitous in healthy adults^6^, is stable over time during life^18^, is associated with human microbiome diversity and high gene count^10,11^, and harbors specific features that suggest its coevolution as a commensal^13^, with specific interactions with host mucosal immunity^46^. In addition, increasing evidence has been reported for innate and adaptative immune recognition and activation by human-associated archaea, notably by archaeosomes^15^. Association with the absence of obesity (Fig. 1)^10,11^ further suggests that *M*. *smithii* is a critical commensal for human health^7^.

Only a few teams in the world have reported the successful culture of human archaea in the literature (Supplementary Tables S1 and S2). Working on microbiota and malnutrition^12,47–49^, we recently discovered the critical role of 3 major nonenzymatic human plasmatic antioxidants (ascorbate, glutathione, uric acid) in the culture of anaerobes and Archaea^29,35,37^. Strikingly, these 3 molecules are also the 3 major nonenzymatic antioxidants in human colostrum and milk^50^. This finding is not random and suggests that human methanogenic archaea are dependent on the concentration of these antioxidants in the gut. Further studies are needed to confirm this, but this discovery has been decisive in the successful culture of several archaea from the human microbiome^29^.

We have previously shown that *M*. *smithii* was associated with the absence of obesity^51^ and malnutrition^12^. In the literature, there has been a general confusion in the association between methanogenic archaea and obesity^15^. Inconclusive or contradictory results are obtained when different taxonomic levels (*M*. *smithii*^51–53^, *Methanobacteriales*^54,55^, Archaea^56^), breath methane production^56–58^, and overweight and obese individuals^59^ are evaluated together. We previously clarified this issue by a meta-analysis including only comparisons of the frequency of detection of *Methanobrevibacter* species between obese individuals and controls^60^. As a new study has been published^61^, we performed this meta-analysis again and confirmed the association between a decreased frequency of *M*. *smithii* and *Methanobrevibacter* species in obesity (Fig. 1). This consistent effect was not observed with lower taxonomic levels (*Methanobacteriales*, Archaea) and with breath methane production. Accordingly, a genus- and/or species-specific effect of *M*. *smithii* on weight regulation is likely, as previously reported for *Lactobacillus*^51,52,62,63^. At least two large-scale metagenomics and 16S amplicon sequencing studies corroborate the association of *M*. *smithii* with the lean phenotype^10,11^.

*Methanosphaera stadtmanae* is a representative of another human methanogenic archaeal genus and a member of the *Methanobacteriales* order. *M*. *stadtmanae* was associated with different roles in human health, notably with a severe proinflammatory response (TNF-α, IL-1β) not observed for *M*. *smithii*^15^. This species, which was less frequent than *M*. *smithii* in the human gut (approximately 30% versus >95%^6^), was not detected in human colostrum and milk in our study. Moreover, a 3-fold increase in the abundance of *M*. *stadtmanae* was reported in inflammatory bowel diseases (IBD) compared to that of controls, whereas *M*. *smithii*’s abundance did not differ^64^. This species could explain the discrepancy between studies including only *Methanobrevibacter* species and those including larger taxonomical groups (*Methanobacteriales* order, Archaea) and physiological studies analyzing breath methane. Overall, the role of *M*. *smithii* in the commensal human gut microbiota appears to be the result of evolutionary coadaptation of the human host and this archaeal species^7,13,15^. This is not the case for *M*. *stadtmanae* associated with *in vitro* proinflammatory properties^46^ and inflammatory bowel diseases^64^.

Here, we found that *M*. *smithii* was detectable in 33% of nonobese mothers. *M*. *smithii* was detected less frequently (14%) in colostrum or milk of obese mothers. The difference was not significant. However, a two-fold difference (33 versus 14%) and an Odds ratio of 0.3 should not be neglected even if the p-value is 0.08^65^, which is consistent with the association between *M*. *smithii* in feces and absence of obesity (Fig. 1). Strikingly, *M*. *smithii* was detected more frequently in overweight versus lean mothers. Finally, the normal distribution of maternal BMI observed only when *M*. *smithii* was detected suggested a role of *M*. *smithii* for weight regulation (Fig. 4). To clarify if detection of *M*. *smithii* in colostrum and/or milk is associated with absence of maternal obesity, we calculated that at least 88 cases and 88 controls should be included in a future study to rule out a putative association with a two-sided confidence level 95% and power of 80%.

In conclusion, we showed that human colostrum and milk contain viable *M*. *smithii* and *M*. *oralis*. This supports a key role for the early initiation of breastfeeding and sheds light on the mechanisms underlying the inheritance of methanogenic archaea, specifically *M*. *smithii*, a neglected critical commensal highly suspected to be involved in weight regulation.

## Methods

### Patients and samples

Healthy mothers aged over 18 years of age with a full-term pregnancy who had opted for mixed or exclusive breastfeeding were selected to participate in this study. Breast colostrum and milk samples were collected from healthy women at the second and tenth day postpartum, respectively. The sampling was performed by a pediatrician. Before sampling, the pediatrician washed his or her hands with alcoholic solution and then put gloves on before touching the sampling site. Nipples and areolas were not cleaned. The samples (250–1000 µL) were collected in sterile tubes by manual pressing at the neonatology unit of the Hôpital de la Conception and Hôpital Nord, Marseille, France. The main inclusion criteria of this study were breastfeeding and acceptance of participation in the study protocol. The main criteria for exclusion were exclusively artificial breastfeeding, refusal of participation and the presence of a disorder (mastitis or breast abscess) that may have an impact on the microbiota of the study subject. The data collected from the included mothers were recruitment center, age, weight before pregnancy, height (body mass index before pregnancy was calculated), gestity, parity, gestational diabetes, and tobacco smoking. For pregnancy, we collected gestational age, preterm delivery, delivery route (vaginal or C-section) and twin pregnancy. For newborns, we collected sex, birth weight, birth height, head circumference, exclusive or mixed breastfeeding. Body mass index, weight-for-length z-score, weight-for-age z-score, length-for-age z-score, BMI-for-age z-score, and head-circumference-for-age z-score were calculated using the software WHO Anthro (version 3.2.2, January 2011) available online (https://www.who.int/childgrowth/software/en/). All participants were informed and gave their written consent before the samples were collected. The ethics committee of the “Institut Fédératif de Pathologies Transmissibles et Pathologies Infectieuses Tropicales 48” approved the consent and study protocol under number 2016–004. The authors certified that this study was not in opposition to the Declaration of Helsinki and was in accordance with French laws (certificates available on request).

### Culture and isolation

A volume of 250 µL of breast milk or colostrum sample was seeded in ambient air in a sterile Hungate tube (Dominique Dutscher, Brumath, France). The culture and isolation of methanogenic archaea were performed according to the previously published protocol^29^ under aerobic conditions using coculture with *Bacteroides thetaiotaomicron*. Each Hungate tube contained 5 mL of SAB broth supplemented with ascorbic acid (1 g/L; VWR International, Leuven, Belgium), uric acid (0.1 g/L) and glutathione (0.1 g/L; Sigma-Aldrich, Saint-Quentin Fallavier, France). The pH of the culture media was adjusted to 7.5 with KOH (10 M). Five milliliters of SAB medium and 250 µL of milk or colostrum were inoculated with *B*. *thetaiotaomicron* (10^5^ cells/mL) for hydrogen production at 37 °C with agitation for seven days. The growth of all methanogens was inferred from the production of methane (CH_4_) detected by gas chromatography, as previously described^66^. Subcultures were seeded on a Petri dish containing SAB medium supplemented with 15 g/L agar and deposited in the upper chamber of a double chamber. A tube containing noninoculated SAB medium was used as a negative control. For solid medium culture, a noninoculated agar Petri dish was used as a negative control.

### DNA extraction and 16S rRNA gene sequencing for colony identification

The identification of colonies obtained with the method described above was confirmed by the following DNA extraction and sequencing protocol. DNA was extracted using the E.Z.N.A.® Tissue DNA Kit (OMEGA Bio-tek, Norcross, GA, USA) according to the manufacturer’s instructions and the modified extraction protocol described by Dridi *et al*.^6^. PCR was performed with a PTC-200 automated thermal cycler (MJ Research, Waltham, USA) in 50 µL of PCR mixture. The archaeal 16S rRNA gene was amplified using a 40-cycle program with the archaeal primers SDArch0333aS15 (50-TCCAGGCCCTACGGG-30) and SDArch0958aA19 (5′-YCCGGCGTTGAMTCCAATT-3′) (Eurogentec, Seraing, Belgium). PCR products were purified and sequenced using a 3500xL genetic analyzer (ThermoFisher, Waltham, MA USA) and a Big-Dye Terminator, version 1.1, cycle sequencing kit DNA according to the manufacturer’s instructions (Applied Biosystems, Foster City, USA). The chromas Pro1.34 software (Technelysium Pty. Ltd) was used for sequence correction. BLASTn (nucleotide Basic Local Alignment Search Tool) searches were performed against GenBank (http://blast.ncbi.nlm.nih.gov.gate1.inist.fr/Blast.cgi) for taxonomic assignment. Two negative control samples consisting of master mix and RNase-free water were introduced for every 5 samples tested.

### Genome sequencing

Genomic DNA of the isolates C2 CSUR P5816 and M2 CSUR P5920 cultured from colostrum and milk, respectively, were sequenced using MiSeq Technology (Illumina, Inc., San Diego CA 92121, USA) with a paired-end and barcode strategy with 15 other projects constructed according to the Nextera XT library kit (Illumina). The gDNA was quantified by a Qubit assay with a high-sensitivity kit (Life Technologies, Carlsbad, CA, USA) to 0.8 ng/µL and diluted to require one ng of DNA as input. The “tagmentation” step fragmented the genomic DNA. Then, PCR cycle amplification completed the tag adapters and introduced the dual-index barcodes. After purification on AMPure beads (Life Technologies), the libraries were normalized on specific beads according to the Nextera XT protocol (Illumina). Normalized libraries were pooled into a single library for sequencing via MiSeq. A pooled single-strand library was loaded onto the reagent cartridge and then onto the instrument along with the flow cell. We performed automated cluster generation and paired-end sequencing with dual-index reads in a single 39-hour run in 2 × 251-bp. We obtained total information with an 8.5 gigabase sequence with an 899 K/mm2 cluster density, with 94.9% (16,382,000 clusters) of the clusters passing quality control filters. Within this pooled run, the index representation of the isolate was determined to be 13.50%. The 2,212,330 paired-end reads were filtered according to the read qualities.

### Genome assembly and construction of the rhizome

The Illumina reads obtained for both strains were assembled using SPAdes software (http://bioinf.spbau.ru/spades) helped by GapFiller v2.1.1 35 to reduce the set. Subsequently, the assembly was refined using manual finishing. BLASTp was performed on all translated coding sequences using the nr database. For each coding DNA sequence, the best BLAST hit was determined from the max bit score. Only all hits related to the *Methanobrevibacter* genus after data filtering were considered ORFans. The origins of all genes of the two strains C2 CSUR P5816 (*M*. *smithii*) and M2 CSUR P5920 (*M*. *oralis*) were determined according to their taxonomic affiliation. The rhizome representation was created using Circos software 36 for both strains (*M*. *oralis* M2 CSUR P5920 and *M*. *smithii* C2 CSUR P5816), as well as the *M*. *oralis* strain MBORA DSM 7256 isolated from the human mouth^23^ and *M*. *smithii* strain WWM1085 isolated from the human gut^24^.

### Detection of *M*. *smithii* and *M*. *oralis* using qPCR

Following the culture and characterization of methanogenic archaeal strains obtained after direct inoculation of fresh samples from 13 mothers, we sought to estimate the proportion of archaea in the colostrum and/or milk of the whole cohort by real-time PCR. DNA was extracted using the E.Z.N.A.® Tissue DNA Kit (OMEGA Bio-tek) according to the manufacturer’s instructions and the modified extraction protocol described by Dridi *et al*.^6^. Real-time PCR assays were performed with an MX3000TM system (Stratagene, Amsterdam, The Netherlands) using the QuantiTect Probe PCR Kit (Qiagen, Courtaboeuf, France) with 5 pmol of each primer, a probe labeled with FAM, and 5 mL of DNA in a final volume of 25 mL. The PCR amplification program for *M*. *smithii* was 95 °C for 15 min, followed by 42 cycles of 95 °C for 30 s and 60 °C for 1 min, and that for *M*. *oralis* amplification was 95 °C for 15 min, followed by 42 cycles of 95 °C for 10 s, 60 °C for 45 s and 45 °C for 30 s as previously described^6,67^. The primers and probes used for *M*. *oralis* and *M*. *smithii* amplification were as follows: *M*. *oralis*: M-cnp602F 5′-GCTGGTGTAATCGAACCTAAACG-3′, cnp602R 5′-CACCCATACCCGGATCCATA-3′ FAM 5′-AGCAGTGCACCTGCTGATATGGAAGG-3′; *M*. *smithii*: Smit.16S-740F, 5′-CCGGGTATCTAATCCGGTTC-3′, Smit.16S-862R, 5′-CTCCCAGGGTAGAGGTGAAA-3′, Smit.16S FAM 5′-CCGTCAGAATCGTTCCAGTCAG-3′. We used calibration curves as previously described^6,67^. Two negative control samples consisting of master mix and RNase-free water were introduced for every 5 samples tested.

### Metabolic variables and groups

As we previously associated human methanogenic archaea and specifically *M*. *smithii* with the absence of obesity (Fig. 1), we sought to test whether this association was found in the present study. A body mass index (BMI) categorical variable was defined as follows: underweight (BMI <18.5), lean (normal weight, BMI ≥18.5 and ≤25), overweight (BMI >25 and <30) and mothers who met obesity criteria (BMI ≥30).

### Statistical analysis

We compared the clinical characteristics of mothers and newborns according to the detection of *M*. *smithii* in colostrum and/or milk. When comparing newborn characteristics, twins were excluded. Quantitative variables were analyzed using the unpaired t-test or Mann-Whitney test according to the distribution of the data. Qualitative variables were analyzed using the two-sided Fisher or mid-p exact test. The test used to test the previously reported association between the depletion of *M*. *smithii* and obesity (Fig. 1) was unilateral. A p-value < 0.05 was considered significant. Comparison of the distribution of maternal BMI values between mothers with or without detection of *M*. *smithii* was performed by observation of density histograms, Kolmogorov-Smirnov normality test, Levene’s test and measure of skewness. GraphPad Prism v8.1.1 (GraphPad software, San Diego, CA USA) and XLSTAT 2019.1.2 (Addinsoft, Paris, France) were used for statistical analysis.

### Accession codes

The genome and 16S sequences of *M*. *smithii* strain C2 CSUR P5816 were deposited in EMBL-EBI under the accession numbers CAABOX 000000000 and LR590664, respectively. The genome and 16S sequences of *M*. *oralis* strain M2 CSUR P5920 were deposited in EMBL-EBI under the accession numbers OKQL00000000 and LR590665, respectively. The partial 16S rRNA gene sequences of the 7 other *M*. *smithii* strains C1 CSUR P5920, M1 CSUR P5819, M6 CSUR P5818, C3 CSUR P5922, M7 CSUR P5820, M5 CSUR P5919 and M3 CSUR P5921 were deposited under the Bioproject PRJEB32060 and numbered from LR584035 to LR584041.

## Supplementary information

Supplementary Data

Dataset 1
