# Supplementary material for: Culture of Methanogenic Archaea from Human Colostrum and Milk
Source: Sci Rep. 2019 Dec 9;9:18653. doi: 10.1038/s41598-019-54759-x (PMC6901439; doi:10.1038/s41598-019-54759-x)
Supplement: Supplementary file 1 — Supplementary Data [file 41598_2019_54759_MOESM1_ESM.docx]

**Culture of Methanogenic Archaea from Human Colostrum and Milk**

Amadou Hamidou TOGO, Ghiles GRINE, Saber KHELAIFIA, Clothilde DES ROBERT, Véronique BREVAUT, Aurelia CAPUTO, Emeline BAPTISTE, Marion BONNET, Anthony LEVASSEUR, Michel DRANCOURT, Matthieu MILLION, Didier RAOULT

**SUPPLEMENTARY DATA**

**Supplementary Figure 1.** **Culture of *Methanobrevibacter smithii* and *Methanobrevibacter oralis* from Human Colostrum and Milk on agar plate**

**
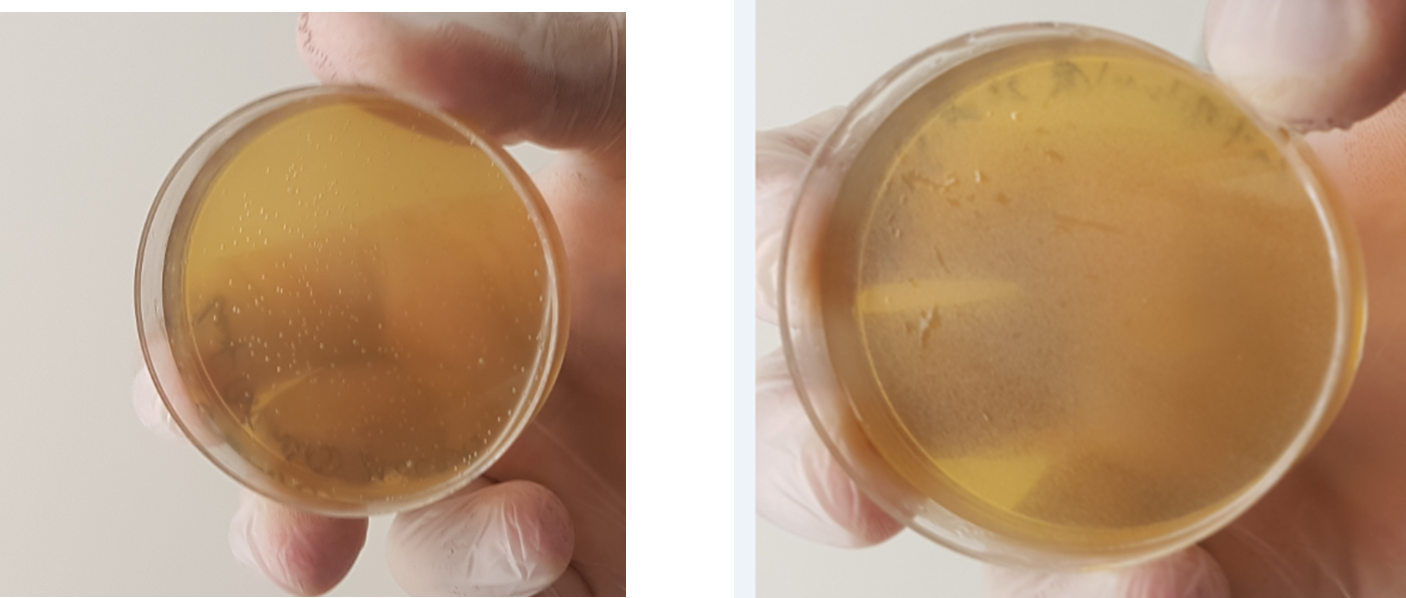

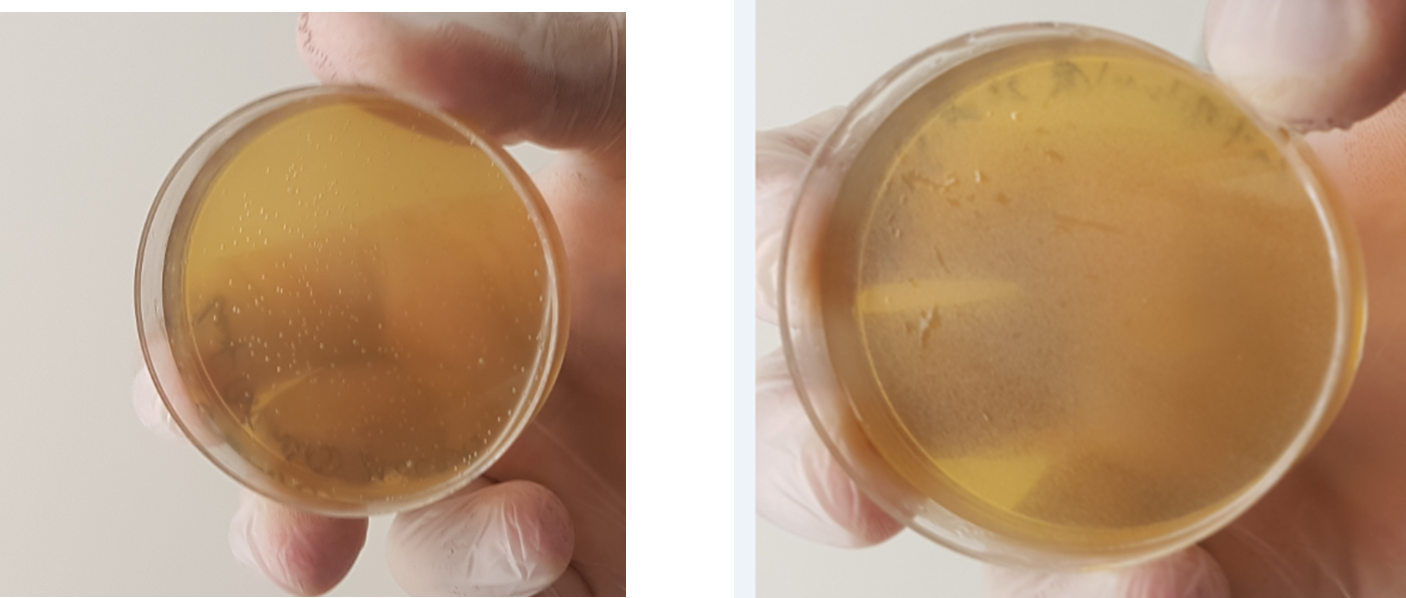
**

***Methanobrevibacter oralis***

***Methanobrevibacter smithii***

**Supplementary Figure 2. Quantification of *Methanobrevibacter smithii* cells in human colostrum and milk based on quantitative PCR**

**
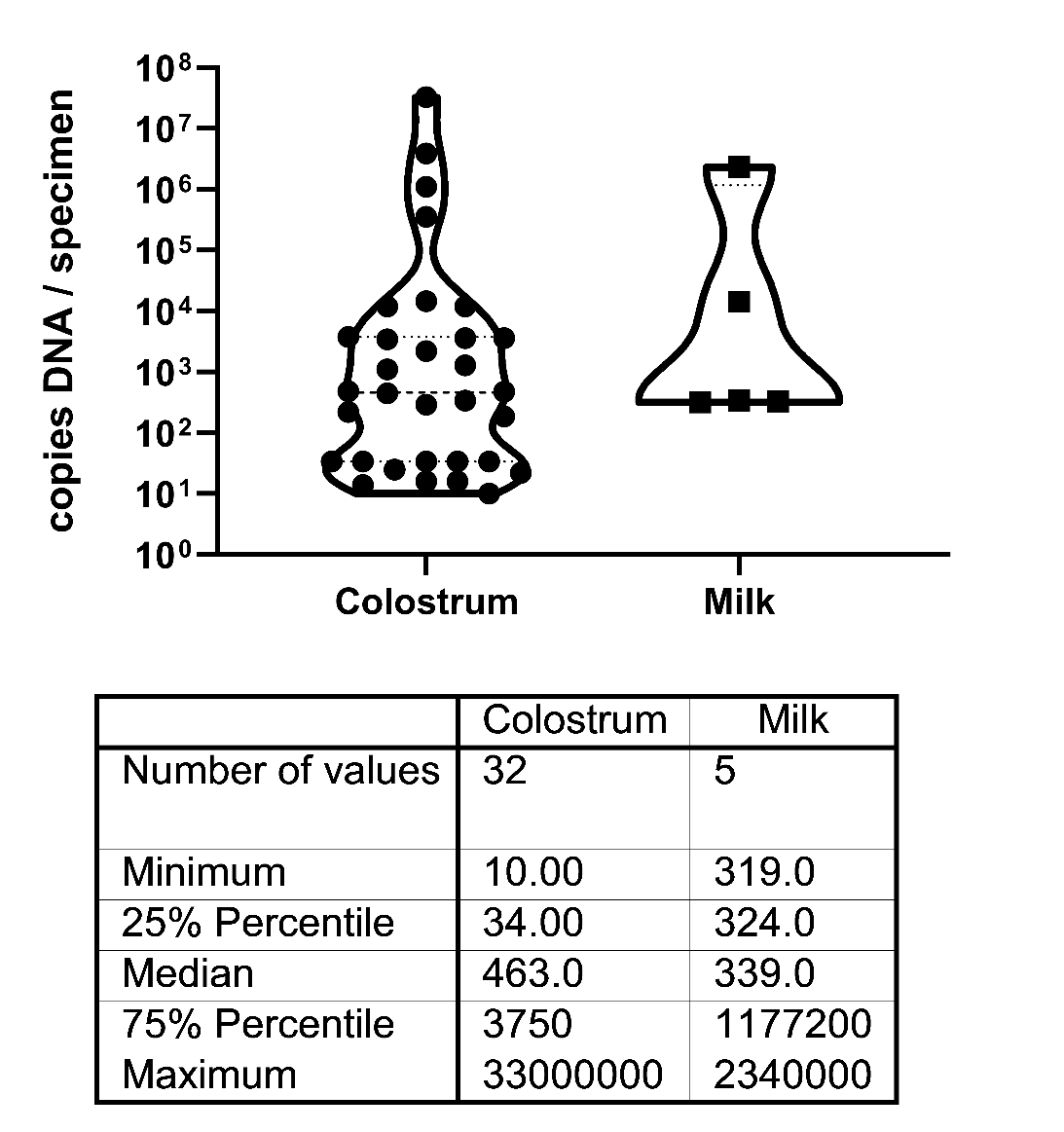
**

**Supplementary Table S1. Studies reporting the culture of archaea in humans from other centers**

| **Reference** | **Species** | **Anatomical site** |
| --- | --- | --- |
| Miller, 1982^1^ | *Methanobrevibacter smithii** | Feces |
| Miller, 1982^2^ | *Methanobrevibacter smithii* | Feces |
| Miller, 1983^3^ | *Methanobrevibacter smithii* | Feces |
| Miller, 1984^4^ | *Methanobrevibacter smithii* | Colon segment isolated from the normal fecal stream |
| Miller, 1983^5^  Miller, 1985^6^ (same study) | *Methanosphaera stadtmanae*,*** | Feces |
| Weaver, 1986^7^ | *Methanobrevibacter smithii* | Feces (tap water enema) |
| Ferrari, 1994^8^ | *Methanobrevibacter oralis*,*** | Subgingival samples of healthy subjects |
| Hansen, 2011^9^ | *Methanobrevibacter smithii* | Feces |
| Jennings, 2017^10^ | *Methanobrevibacter smithii* | Feces |

*First isolation in humans. **New species (first isolation of this species from human samples).

**Supplementary Table S2. Studies reporting the culture of archaea in humans from our center**

| **Reference** | **Species** | **Anatomical site** |
| --- | --- | --- |
| Dridi, 2012^11^ | *Methanomassiliicoccus luminyensis*,*** | Feces |
| Huynh, 2015^12^ | *Methanobrevibacter oralis*  *Methanobrevibacter smithii* | Periodontitis |
| Khelaifia, 2013^13^  Khelaifia, 2014^14^ | *Methanobrevibacter smithii*  *Methanobrevibacter millerae**  *Methanobrevibacter arboriphilicus** | Feces |
| Khelaifia, 2016^15^ | *Methanobrevibacter oralis*  *Methanobrevibacter smithii* | Oral cavity  Feces |
| Nkamga, 2016^16^ | *Methanobrevibacter smithii* | Paravertebral abscess |
| Huynh, 2017^17^ | *Methanobrevibacter massiliense*,*** | Periodontitis |
| Drancourt, 2017^18^ | *Methanobrevibacter oralis* | Brain abscess |
| Grine, 2017^19^ | *Methanobrevibacter smithii* | Stomach of newborns |
| Grine, 2018^20^ | *Methanobrevibacter oralis*  *Methanobrevibacter smithii* | Saliva |
| Seck, 2017^21^ | *Haloferax alexandrines**  *Haloferax massiliensis*,*** | Feces |

*First isolation in humans. **New species (first isolation of this species from human samples).

**Table 3. Comparison of clinical characteristics of mothers and newborns according to the detection of *M. smithii* in colostrum and/or milk**

|  | **Positive** | **Negative** | **p-value** |
| --- | --- | --- | --- |
|  |  |  |  |
| **Mother** | n = 40 | n = 88 |  |
| Recruitment center (Hôpital Conception) | 32 (80.0%) | 69 (78.4%) | 0.84^a^ |
| Mother age | 30.7 ± 6.9 | 30.2 ± 6.1 | 0.65^b^ |
| Mother weight (kg) | 65 [58 - 73] | 63 [54 - 74] | 0.49^c^ |
| Mother height (cm) | 163.7 ± 6.1 | 162.9 ± 6.3 | 0.51^b^ |
| Mother BMI | 24.0 [21.6 - 26.4] | 23.3 [20.6 - 27.6] | 0.59^c^ |
|  |  |  |  |
| Underweight (BMI < 19) | 2 (5.0%) | 5 (5.6%) | >0.99^d^ |
| Lean (BMI ≥ 19 and ≤ 25) | 21(52%) | 53 (60.2%) | 0.41^a^ |
| Overweight (BMI > 25 and < 30) | 15 (37.5%) | 18 (20.4%) | 0.047^a^ |
| Obese (BMI ≥ 30) | 2 (5.0%) | 12 (13.6%) | 0.08^e^ |
|  |  |  |  |
| Gestity | 2 [1 - 4] | 2 [2 - 4] | 0.85^c^ |
| Parity | 1 [0 - 2] | 1 [0 - 2] | 0.91^c^ |
| Gestational diabetes | 8 (20.0%) | 11 (12.5%) | 0.27^a^ |
| Smoking (Y/N) | 7 (17.5%) | 9 (10.2%) | 0.25^a^ |
|  |  |  |  |
| **Pregnancy** |  |  |  |
| Gestational age (WA) | 40 [39 - 41] | 39 [38 - 40] | 0.41^c^ |
| Preterm (below 37 WA) | 4 (10.0%) | 6 (6.8%) | 0.76^f^ |
| Cesarean section | 13 (32.5%) | 20 (22.7%) | 0.24^a^ |
| Twin pregnancy | 1 (2.5%) | 3 (3.4%) | >0.99^f^ |
|  |  |  |  |
| **Newborn (twins excluded)** | n = 39 | n = 85 |  |
| Male | 16 (41.0%) | 43 (50.6%) | 0.32^a^ |
| Birth weight (kg) | 3249 ± 620 | 3145 ± 524 | 0.33^b^ |
| Birth height (cm) | 49.0 [47.0 – 50.0] | 48.50 [47.0 – 50.0] | 0.55^c^ |
| Body mass index | 13.4 [12.3 - 15.2] | 13.4 [12.6 - 14.7] | 0.60^c^ |
| Head circumference (cm) | 34.6 ± 1.5 | 34.3 ± 1.7 | 0.25^b^ |
| Exclusive breastfeeding | 29 (74.3%) | 68 (80.0%) | 0.48^a^ |
|  |  |  |  |
| **Comparison to WHO standard curves^g^** | **n = 36** | **n = 80** |  |
| Weight-for-length z-score | 0.56 [-0.92 to 1.43] | 0.32 [-0.43 to 1.14] | 0.93^c^ |
| Weight-for-age z-score | -0.02 ± 1.27 | -0.23 ± 1.03 | 0.34^b^ |
| Length-for-age z-score | -0.37 ± 1.19 | -0.49 ± 1.19 | 0.62^b^ |
| BMI-for-age z-score | 0.02 [-0.91 to 1.36] | 0.10 [-0.55 to 1.07] | 0.78^c^ |
| BMI-for-age z-score > +2 | 3 (8.3%) | 1 (1.2%) | 0.18^f^ |
| Head-circumference-for-age z-score | 0.45 ± 1.22 | 0.20 ± 1.26 | 0.65^b^ |

BMI: body mass index; WA: weeks of amenorrhea. ^a^Two-sided chi square test, ^b^Two-sided unpaired t-test, ^c^Two-sided unpaired Mann-Whitney test, ^d^Two-sided exact Fisher test, ^e^One-sided mid-p exact test (obesity previously associated with depletion of *M. smithii*^11,12^, see main text), ^f^Two-sided Fisher exact test, ^g^Twins and preterm excluded.

**References**

1. Miller, T. L., Wolin, M. J., Conway de Macario, E. & Macario, A. J. Isolation of *Methanobrevibacter smithii* from human feces. *Applied and Environmental Microbiology* **43**, 227-232 (1982).
2. Miller, T. L. & Wolin, M. J. Enumeration of *Methanobrevibacter smithii* in human feces. *Arch Microbiol* **131**, 14-18 (1982).
3. Miller, T. L. & Wolin, M. J. Stability of *Methanobrevibacter smithii* populations in the microbial flora excreted from the human large bowel. *Appl Environ Microbiol* **45**, 317-318 (1983).
4. Miller, T. L., Weaver, G. A. & Wolin, M. J. Methanogens and anaerobes in a colon segment isolated from the normal fecal stream. *Appl Environ Microbiol* **48**, 449-450 (1984).
5. Miller, T. L. & Wolin, M. J. Oxidation of hydrogen and reduction of methanol to methane is the sole energy source for a methanogen isolated from human feces. *J Bacteriol* **153**, 1051-1055 (1983).
6. Miller, T. L. & Wolin, M. J. *Methanosphaera stadtmaniae* gen. nov., sp. nov.: a species that forms methane by reducing methanol with hydrogen. *Arch. Microbiol.* **141**, 116-122 (1985).
7. Weaver, G. A., Krause, J. A., Miller, T. L. & Wolin, M. J. Incidence of methanogenic bacteria in a sigmoidoscopy population: an association of methanogenic bacteria and diverticulosis. *Gut* **27**, 698-704 (1986).
8. Ferrari, A., Brusa, T., Rutili, A., Canzi, E. & Biavati, B. Isolation and characterization of *Methanobrevibacter oralis* sp. nov. *Current Microbiology* **29**, 6 (1994).
9. Hansen, E. E. *et al.* Pan-genome of the dominant human gut-associated archaeon, *Methanobrevibacter smithii*, studied in twins. *Proc Natl Acad Sci U S A* **108 Suppl 1**, 4599-4606, doi:10.1073/pnas.1000071108 (2011).
10. Jennings, M. E., Chia, N., Boardman, L. A. & Metcalf, W. W. Draft Genome Sequence of *Methanobrevibacter smithii* isolate WWM1085, obtained from a human stool sample. *Genome Announc* **5**, doi:10.1128/genomeA.01055-17 (2017).
11. Dridi, B., Fardeau, M.-L., Ollivier, B., Raoult, D. & Drancourt, M. *Methanomassiliicoccus luminyensis* gen. nov., sp. nov., a methanogenic archaeon isolated from human faeces. *International Journal of Systematic and Evolutionary Microbiology* **62**, 1902-1907, doi:10.1099/ijs.0.033712-0 (2012).
12. Huynh, H. T., Pignoly, M., Nkamga, V. D., Drancourt, M. & Aboudharam, G. The repertoire of archaea cultivated from severe periodontitis. *PLoS One* **10**, e0121565, doi:10.1371/journal.pone.0121565 (2015).
13. Khelaifia, S., Raoult, D. & Drancourt, M. A versatile medium for cultivating methanogenic Archaea. *PLoS ONE* **8**, e61563, doi:10.1371/journal.pone.0061563 (2013).
14. Khelaifia, S., Garibal, M., Robert, C., Raoult, D. & Drancourt, M. Draft genome sequence of a human-associated isolate of *Methanobrevibacter arboriphilicus*, the lowest-G+C-content archaeon. *Genome Announc* **2**, doi:10.1128/genomeA.01181-13 (2014).
15. Khelaifia, S. *et al.* Aerobic culture of methanogenic archaea without an external source of hydrogen. *European Journal of Clinical Microbiology & Infectious Diseases* **35**, 985-991, doi:10.1007/s10096-016-2627-7 (2016).
16. Nkamga, V. D., Lotte, R., Roger, P. M., Drancourt, M. & Ruimy, R. *Methanobrevibacter smithii* and *Bacteroides thetaiotaomicron* cultivated from a chronic paravertebral muscle abscess. *Clinical microbiology and infection : the official publication of the European Society of Clinical Microbiology and Infectious Diseases* **22**, 1008-1009, doi:10.1016/j.cmi.2016.09.007 (2016).
17. Huynh, H. T. T., Pignoly, M., Drancourt, M. & Aboudharam, G. A new methanogen "*Methanobrevibacter massiliense*" isolated in a case of severe periodontitis. *BMC Res Notes* **10**, 657, doi:10.1186/s13104-017-2980-3 (2017).
18. Drancourt, M. *et al.* Evidence of archaeal methanogens in brain abscess. *Clinical infectious diseases : an official publication of the Infectious Diseases Society of America* **65**, 1-5, doi:10.1093/cid/cix286 (2017).
19. Grine, G., Boualam, M. A. & Drancourt, M. *Methanobrevibacter smithii*, a methanogen consistently colonising the newborn stomach. *European Journal of Clinical Microbiology & Infectious Diseases* **36**, 2449-2455, doi:10.1007/s10096-017-3084-7 (2017).
20. Grine, G. *et al.* Tobacco-smoking-related prevalence of methanogens in the oral fluid microbiota. *Sci Rep* **8**, 9197, doi:10.1038/s41598-018-27372-7 (2018).
21. Seck, E. H. *et al.* Salt in stools is associated with obesity, gut halophilic microbiota and Akkermansia muciniphila depletion in humans. *Int J Obes (Lond)*, doi:10.1038/s41366-018-0201-3 (2018).
